# The prevalence and genotype distribution of high-risk human papillomaviruses among women in Xianning, China

**DOI:** 10.1186/s12985-024-02413-y

**Published:** 2024-06-18

**Authors:** Bin Qiu, Na Jiang, Jinpeng Jiang, Xuebao Mao, Xiuhong Wang

**Affiliations:** Department of Gynecology, Xianning Central Hospital, The First Affiliated Hospital of Hubei University of Science and Technology, Xianning, Hubei 437000 China

**Keywords:** Human papillomavirus, Genotypes, Cervical cancer, Cervical lesions, Vaccines, China

## Abstract

**Background:**

The persistent infection of high-risk Human papillomavirus(HPV) is considered the main cause of cervical intraepithelial neoplasia and cervical cancer. But various cervical lesions caused by HPV infection can be properly prevented by timely vaccination. However, the distribution of HPV genotypes varies geographically.

**Methods:**

Retrospective analysis of high-risk HPV prevalence of 16,150 women from 2020 to 2022 in xianning of China. HPV genotyping was performed using a PCR-RDB Kit that can detect 18 high-risk HPV genotypes recommended by China’s National Medical Products Administration. The prevalence of 18 high-risk HPV genotypes and their relationship with cervical lesions as well as vaccine efficacy were analyzed.

**Results:**

A total of 2431 women were confirmed to have different types of high-risk HPV infections. The overall positive rate reached 15.05%(2431/16,150). The most prevalent high-risk HPV genotypes were HPV52, 16, 58, 53, and 51. The prevalence of high-risk HPV reached peak at age ≤ 20(20.95%) and age ≥ 61(20.56%). The most prevalent high-risk HPV genotypes were HPV16, 58, 18, 33 and 52 in cervical cancer cases, HPV16, 52, 58, 33 and 18 in CIN2/3 cases, and HPV52, 58, 16, 53 and 18 in CIN1 cases, respectively.

**Conclusion:**

HPV16, 58 and 18 are the most dangerous and carcinogenic genotypes in xianning, China. Conducting epidemiological investigations on high-risk HPV has significant clinical value in guiding HPV vaccination work.

## Introduction

Cervical cancer is a common malignancy in women with a high morbidity and mortality. Although cervical screening methods is increasingly advanced, the incidence of cervical cancer is still exceeds 500,000 every year in the world; cervical cancer remains a major challenge for global public health [1, 2]. It is the 4th female carcinoma globally, and there are about 80% of patients with cervical carcinoma in developing countries due to failed early-screening for cervical carcinoma [3]. As everyone knows, China is the largest developing country and also the second most populous country in the world. Cervical cancer is the second most common cancers among Chinese women, and the incidence and mortality rates are still increasing [4]. Currently, the popularity rate of cervical screening programs in China still needs to be improved. Human papillomaviruses (HPV) are small double stranded DNA viruses that belong to the family of papillomavirus [5]. So far, scientists have discovered over 200 HPV genotypes. Generally, HPVs are classified as high-risk HPV genotypes which are closely associated with cervical cancers, and low-risk HPV genotypes which are not carcinogenic to humans [6]. Over 80% of women are infected with HPV throughout their lifetime, however, 90% of these infections can naturally clear within 2 years [7]. Significantly, the persistent infection of high-risk HPV is considered the main cause of cervical intraepithelial neoplasia (CIN) and cervical cancer [8, 9]. According to global data, the most prevalent high-risk HPV genotypes are HPV16, 18, 33, 45 and 31 in cervical cancer patients, while HPV16, 18, 31, 58 and 52 in the general population [10]. However, the distribution of HPV genotypes varies geographically [11, 12]. Due to the large population and vast geographical area, the prevalence of HPV also varies in China. Fortunately, cervical cancer can be largely prevented through vaccines. The efficacy and safety of the HPV vaccines in preventing cervical cancer have been extensively confirmed by clinical data [13–17]. The main components of HPV vaccines are virus-like particles(VLPs) derived from the L1 proteins [18]. At present, the bivalent (includes HPV16 and 18 VLPs), quadrivalent (includes HPV6, 11, 16 and 18 VLPs) and 9-valent (includes HPV6, 11, 16, 18, 31, 33, 45, 52 and 58 VLPs) HPV vaccines are available [19, 20]. Choosing the right vaccine is the primary condition to ensure vaccination efficiency and is key in helping reduce cervical cancer. However, limited data on HPV prevalence in Central China women are available so far, especially in Hubei Province. Our study evaluated the characteristics of 18 high-risk HPVs distribution among women in Xianning, Hubei Province, and can serve as a reference for improving local vaccination strategies.

## Methods

### Data source

A total of 16,150 data on HPV prevalence from women who participated in cervical cancer screening (HPV and cytology) in obstetrics and gynecology outpatient clinics and health examination center were collected from 2020 to 2022 in Xianning Central Hospital. The data was in the form of 10-year age groups. The average age of the study population was 46.2 years (range 18–80 years). The participants were enrolled according to the following selection criteria: was willing to undergo HPV testing; sexually active; non-pregnant; were not being treated with vaginal medicine in the past week. Women with abnormal cervical cancer screening voluntarily undergo cervical biopsy. A total of 688 HPV data were collected from women who underwent cervical biopsy and were diagnosed with cervical lesions.

### Ethical standards

The authors assert that this study comply with the World Medical Association Declaration of Helsinki. The Scientific and Ethical Committee of Xianning Central Hospital approved the study protocols. Data was analyzed anonymously.

### HPV genotyping

HPV genotyping was performed using a PCR-RDB Kit provided by HEAS BioTech Co., Ltd (Guangzhou, China) that can detect 18 high-risk HPV genotypes (16, 18, 26, 31, 33, 35, 39, 45, 51, 52, 53, 56, 58, 59, 66, 68, 73 and 82) recommended by China’s National Medical Products Administration. All detection procedures were executed in accordance with the instructions provided by the manufacturer.

### Pathological diagnosis

Pathological identifications were performed in conformity with the 2014 World Health Organization (WHO) (Fourth Edition) classification criteria. Cervical lesions are classified by pathological grade and described as follows: low-grade intraepithelial neoplasia (CIN1), high-grade intraepithelial neoplasia (CIN2 and CIN3), and cervical cancer (CC). We grouped women with CIN2 and CIN3 as CIN2/3.

### Statistical analysis

All statistical analyses were carried out using SPSS 22.0 software. Pearson’s χ2 test was performed to evaluate the significance of differences between designated groups. Two-sided P values were obtained in all analyses and interpreted as being significant when P was less than 0.05(*P* < 0.05). Multiple infections were added to single genotypes in accordance with a proportional weighting attribution. For example, if three CC lesions found to test positive for both HPV 16 and 33, and there were 9 cases infected by HPV 16 as a single type and 1 case infected by HPV 33 as a single type, then [3*9/(9 + 1)] or 2.7 of these two multi-type infected lesions would be attributed to HPV 16 and [3*1/(9 + 1)] or 0.3 attributed to HPV 33.

## Results

### HPV infection in the study population

A total of 16,150 samples were collected for HPV infection detection in Xianning during 2020–2022. The results demonstrated that 2431 cases were high-risk HPV positive, and the overall prevalence was 15.05%. The infection rate decreased from 20.95% in women aged ≤ 20 years to 13.43% in those aged 41–50 years, and then increased to 20.56% in those aged ≥ 61 years. The difference between the groups was statistically significant (*P* < 0.01) (Table 1).

**Table 1:** Age-specific results of HPV genetyping in 16,150 women

Age group (y)	Total(*n*)	Positive(*n*)	Negative(*n*)	Positive rate(%)
≤ 20	105	83	22	20.95
21**~**30	2788	2372	416	14.92
31**~**40	4516	3866	650	14.39
41**~**50	5324	4609	715	13.43
51**~**60	2595	2136	459	17.69
≥ 61	822	653	169	20.56
Total	16,150	13,719	2431	15.05

### HPV genotype distribution in positive population

Among 2431 high-risk HPV positive samples, 1896 cases (77.99%) were single infections and 535 cases (22.01%) were multiple infections. As shown in Table 2, the five most prevalent genotypes were HPV52 (21.81%), followed by HPV16 (16.69%), HPV58 (10.98%), HPV53 (8.45%) and HPV51 (6.37%).

**Table 2:** HPV genotype distribution in positive population

HPV genotype	Single(*n* = 1896)	Multiple(*n* = 1227)	Total(*n* = 3123)	Positive rate(%)
16	360	161	521	16.69
18	89	46	135	4.32
26	5	11	16	0.51
31	59	58	117	3.75
33	93	73	166	5.32
35	29	31	60	1.92
39	68	51	119	3.81
45	22	12	34	1.09
51	113	86	199	6.37
52	443	238	681	21.81
53	172	92	264	8.45
56	67	63	130	4.16
58	209	134	343	10.98
59	30	26	56	1.79
66	58	51	109	3.49
68	59	68	127	4.07
73	5	10	15	0.48
82	15	16	31	0.99

### HPV infection in cervical lesions

A total of 688 HPV data of cervical lesions were collected. Among them, there were 124 cases of CIN1, 417cases of CIN2/3, and 147 cases of cervical cancer. The high-risk HPV positive rate in patients with CIN1, CIN2/3, and CC were 79.03%(98/125), 93.29%(389/417) and 95.92%(141/147), respectively. Among 628 high-risk HPV positive cervical lesions, the single infections and multiple infections were 75(76.53%) and 23%23.47% in CIN1 cases, 280%nd 118%83.69% and 23%16.31% in CC cases, respectively. The difference between the groups was statistically significant (*P* < 0.01) (Table 3).

**Table 3:** HPV genetyping in positive cervical lesions

cervical lesions	Single((*n*)	Multiple(*n*)	Total(*n*)	Positive rate(%)
CIN 1	75	23	98	79.03
CIN 2/3	280	109	389	93.29
CC	118	23	141	95.92
Total	473	155	628	91.28

### HPV genotype distribution in positive cervical lesions

As shown in Table 4, the five most prevalent genotypes were HPV16 (33.94%), followed by HPV52 (16.55%), HPV58 (14.98%), HPV33 (6.88%) and HPV18 (6.28%) in cervical lesions, respectively. Among them, the five most prevalent genotypes were HPV16 (55.36%), HPV58 (12.50%), HPV18 (8.93%), HPV33 (6.55%) and HPV52 (4.17%) in CC cases, HPV16 (32.45%), HPV52 (18.79%), HPV58 (16.13%), HPV33 (7.78%) and HPV18 (4.55%) in CIN2/3 cases, and HPV52 (23.31%), HPV58 (13.53%), HPV16 (12.78%), HPV53 (10.53%) and HPV18 (9.77%) in CIN1 cases, respectively.

**Table 4:** HPV genotype distribution in positive cervical lesions [n(%)]

HPV genotype	CIN1(*n* = 133)	CIN2/3(*n* = 527)	CC(*n* = 168)	Total(*n* = 828)
16	17(12.78)	171(32.45)	93(55.36)	281(33.94)
18	13(9.77)	24(4.55)	15(8.93)	52(6.28)
26	0(0.00)	0(0.00)	1(0.60)	1(0.12)
31	6(4.51)	17(3.23)	6(3.57)	29(3.50)
33	5(3.76)	41(7.78)	11(6.55)	57(6.88)
35	4(3.01)	8(1.52)	1(0.60)	13(1.57)
39	1(0.75)	10(1.90)	2(1.19)	13(1.57)
45	0(0.00)	4(0.76)	3(1.79)	7(0.85)
51	13(9.77)	16(3.04)	1(0.60)	30(3.62)
52	31(23.31)	99(18.79)	7(4.17)	137(16.55)
53	14(10.53)	17(3.23)	0(0.00)	31(3.74)
56	3(2.26)	7(1.33)	1(0.60)	11(1.33)
58	18(13.53)	85(16.13)	21(12.5)	124(14.98)
59	2(1.50)	3(0.57)	4(2.38)	9(1.09)
66	2(1.50)	9(1.71)	0(0.00)	11(1.33)
68	3(2.26)	10(1.90)	0(0.00)	13(1.57)
73	0(0.00)	1(0.19)	2(1.19)	3(0.36)
82	1(0.75)	5(0.95)	0(0.00)	6(0.72)

### HPV genotype distribution in positive cervical lesions after proportional weighted

After proportional weighted attribution, the five most prevalent genotypes were HPV16 (64.88%), HPV58 (11.21%), HPV18 (7.72%), HPV33 (5.36%) and HPV31 (3.44%) in CC cases, HPV16 (41.95%), HPV52 (20.21%), HPV58 (18.47%), HPV33 (7.53%) and HPV18 (3.24%) in CIN2/3 cases, and HPV52 (29.40%), HPV58 (13.62%), HPV16 (12.22%), HPV18 (11.27%) and HPV53 (10.15%) in CIN1 cases, respectively (Table 5).

**Table 5:** HPV genotype distribution in positive cervical lesions after proportional weighted [n(%)]

HPV genotype	CIN1(*n* = 98)	CIN2/3(*n* = 389)	CC(*n* = 141)
16	11.98(12.22)	163.17(41.95)	91.48(64.88)
18	11.04(11.27)	12.62(3.24)	10.89(7.72)
26	0.00(0.00)	0.00(0.00)	1.00(0.71)
31	4.87(4.97)	8.39(2.16)	4.85(3.44)
33	4.33(4.42)	29.31(7.53)	7.56(5.36)
35	0.00(0.00)	1.48(0.38)	0.00(0.00)
39	1.00(1.02)	5.66(1.46)	1.04(0.74)
45	0.00(0.00)	0.00(0.00)	3.00(2.13)
51	9.34(9.53)	3.88(1.00)	1.00(0.71)
52	28.81(29.40)	78.62(20.21)	1.62(1.15)
53	9.95(10.15)	1.76(0.45)	0.00(0.00)
56	2.33(2.38)	2.24(0.58)	0.00(0.00)
58	13.35(13.62)	71.84(18.47)	15.80(11.21)
59	0.00(0.00)	2.04(0.52)	1.76(1.25)
66	0.00(0.00)	2.66(0.68)	0.00(0.00)
68	0.00(0.00)	2.30(0.59)	0.00(0.00)
73	0.00(0.00)	0.00(0.00)	1.00(0.71)
82	1.00(1.02)	3.03(0.78)	0.00(0.00)

### HPV vaccine efficacy in cervical lesions

The effective coverage rate of bivalent and quadrivalent vaccines (both include high-risk HPV genotypes 16 and 18 VLPs) were 18.56% in CIN1 cases, 42.16% in CIN2/3 cases, and 69.64% in CC cases. Meanwhile, the effective coverage rate of 9-valent vaccine (includes high-risk HPV genotypes 16, 18, 31, 33, 45, 52 and 58 VLPs) were 59.98% in CIN1 cases, 87.28% in CIN2/3 cases, and 91.97% in CC cases. The effective coverage rate of the 9-valent vaccine was significantly higher than that of the bivalent and quadrivalent vaccines in different degree of cervical lesions ( Table 6).

**Table 6:** HPV vaccine efficacy in cervical lesions [n(%)]

Vaccine type	VLPs	CIN1(*n* = 124)	CIN2/3(*n* = 417)	CC(*n* = 147)
bivalent and quadrivalent	16 and 18	23.02(18.56)	175.79(42.16)	102.37(69.64)
9-valent	16, 18, 31, 33, 45, 52 and 58	74.38(59.98)	363.95(87.28)	135.20(91.97)

## Discussions

Cervical cancer is the most common gynecological malignancy and one of the major cancers threatening women worldwide. According to statistics, approximately 570,000 new cases and 311,000 deaths of cervical cancer are reported worldwide each year [2]; surprisingly, in China alone, the data has reached 106,000 and 48,000 [21] Cervical cancer may be caused by the following factors such as HPV infection, sexual behavior, delivery frequency, smoking, genetics, and immune dysfunction. Among them, the persistent infection of high-risk HPV is considered the main cause of cervical cancer. The HPV represents the most common sexually transmitted infectious agent worldwide. HPV vaccination is a primary preventive measure for preventing and controlling cervical cancer, and the protective effect and safety of the vaccine have been clinically validated [13–17]. In developed countries, due to the popularity of HPV vaccine, the incidence rate as well as mortality of cervical cancer continue to decline. However, HPV vaccination started relatively late in China, coupled with inadequate cervical screening, a growing number of cervical cancer has caused a huge medical burden and economic losses.

Our study demonstrated high-risk HPV prevalence and genotype distribution in xianning, China, with an aim of providing reference data for relevant departments to formulate prevention and control policies for CC in the region. In our study, the positive rate of high-risk HPV was 15.05%, which was very similar to the data of Li H et al. [22], but lower than the data of Zhu et al. [23]. The age-specific positive rate was distributed in a “V” shape. The positive rate decreased from 20.95% in women aged ≤ 20 years to 13.43% in those aged 41–50 years, and then increased to 20.56% in those aged ≥ 61 years, which was similar to the data of Zhu et al. [23]. Young women under the age of 20 are more susceptible to HPV infection due to premature sexual activity, frequent sexual activity, and lack of proper understanding of sexual physiology and hygiene, while older women are mainly affected by decreased hormone levels and immune function. Our study showed that HPV52, 16, 58, 53, and 51 were the five most prevalent genotypes, which was roughly similar to the research results of multiple regions in China [22, 24], but unlike reports from developed countries [25, 26]. As the severity of cervical lesions worsens, the ranking of HPV52 positivity rate continues to decline, reflecting that although HPV52 is most prevalent among Chinese women, its pathogenicity is not as strong as HPV16, 58, and 18.

The results demonstrated that as cervical lesions worsen, the high-risk HPV positive rate gradually increases. The high-risk HPV positive rate in patients with CIN1, CIN2/3, and CC were 79.03%, 93.29% and 95.92%, respectively. All cervical lesion groups were mainly single infections. The five most prevalent genotypes were HPV16, 58, 18, 33 and 52 in CC cases, HPV16, 52, 58, 33 and 18 in CIN2/3 cases, and HPV52, 58, 16, 53 and 18 in CIN1 cases, respectively. It was different from the ranking in the studies of Singh S et al. [27] and Chen W et al. [28], as well as from global data [29], reflecting the regional differences in HPV prevalence. Different high-risk HPV prevalence rates in cervical lesions should be a combination of different cervical cancer screening policy, economic levels, geographic locations, and cultural diversity.

To analyze the relationship between a single HPV gene subtype and cervical lesions, and to calculate vaccine effective coverage, we used proportional weighted attribution analysis for multiple infections. After proportional weighted attribution, the HPV52 dropped out of the top five most prevalent genotypes and was replaced by HPV31 in CC, and the proportion of HPV16 had significantly increased in both CC and CIN2/3. Our data emphasize that HPV16 remained the most dominant strains worldwide. According to WHO data, about 70% of cervical cancer cases are caused by HPV16 and 18 ,which were the most prevalent high-risk HPV genotypes in cervical cancer [29]. In our study, the positive proportion of HPV16 and 18 in cervical cancer was 69.64%, which was close to the global data. It is worth noting that HPV58 ranks in the top three positive rates in CIN1, CIN2/3, and CC, ranking ahead of HPV18, although HPV18 HPV18 has been recognized as the second most prevalent high-risk HPV genotype in the world [30]. However, bivalent and quadrivalent vaccines do not contain HPV58. These data demonstrated that HPV16, 58 and 18 are the most dangerous and carcinogenic genotypes in xianning, China. Through data comparison, it was found that the effective coverage rate of the 9-valent vaccine was significantly higher than that of the bivalent and quadrivalent vaccines in different degree of cervical lesions. Especially in CC and CIN2/3, the effective coverage rates were very high, at 91.97% and 87.28%, respectively. The effective coverage rate of bivalent and quadrivalent vaccines was very low, especially in CINI(18.56%) and CIN2/3(42.16%). Thus, the bivalent vaccines as well as quadrivalent vaccine are not enough to meet the clinical needs, conditional women should receive the 9-valent vaccines as soon as possible. Meanwhile, no vaccine can prevent all high-risk HPV infections, so cervical cancer screening is still necessary after receiving the HPV vaccine.

We are aware that our study has several limitations. Firstly, the data was sourced from One of the cities in Hubei, which means that the representativeness of these results was limited. Secondly, due to the late start of vaccination and the limited coverage of the vaccine, we cannot compare the HPV prevalence between vaccinated and unvaccinated populations. Finally, this is a retrospective study, and we need larger and more specialized prospective studies to further evaluate the relationship between high-risk HPV prevalence and cervical lesions.

In conclusion, the prevalence of high-risk HPV varies among women of different ages and patients with different degree of cervical lesions. High-risk HPV genotype varies geographically. HPV16, HPV58, and HPV18 are the most dangerous and carcinogenic genotypes in xianning, China. Conducting epidemiological investigations on high-risk HPV has significant clinical value in guiding HPV vaccination work.

## Data Availability

The data were collected from Xianning Central Hospital. The data can be freely shared.

## Abbreviations

HPV: Human papillomavirus
CIN: Cervical intraepithelial neoplasia
CC: Cervical cancer
VLPs: Virus-like particles
